# Screening Tool Risk Score Assessment in the Emergency Department for Geriatric (S-TRIAGE) in 28-day mortality

**DOI:** 10.1186/s12245-023-00538-5

**Published:** 2023-09-26

**Authors:** Praphaphorn Supatanakij, Kanruethai Imok, Karn Suttapanit

**Affiliations:** Department of Emergency Medicine, Faculty of Medicine Ramathibodi Hospital, Mahidol University, 270 Rama VI Rd., Ratchathewi, Bangkok, 10400 Thailand

**Keywords:** Mortality, Geriatrics, Triage, Lifesaving intervention

## Abstract

**Background:**

The number of older adults with a high risk of frailty and severe illness continues to increase. Moreover, physiological change and multiple comorbidities are challenging to triage in geriatrics. Therefore, we aimed to evaluate variables to predict 28-day mortality and develop a screening tool to predict mortality and lifesaving intervention among geriatric patients in the emergency department (ED).

**Methods:**

This study was a retrospective, single-center, observational study at the ED of Ramathibodi Hospital, Bangkok. Patients aged ≥ 65 years who visited the ED between January 2018 and December 2019 were enrolled. In the development cohort, univariable logistic regression was used to identify predictors of 28-day mortality in older patients. A predictive model for mortality and the need for lifesaving intervention was developed by multivariable logistic regression. In addition, the score was validated with internal validation and compared between development and validation set by chi-square.

**Results:**

We enrolled 1393 patients. In the development cohort, among these 1002 patients, 103 (10.3%) of whom died within 28 days. Malignancy, shock index (SI), systolic blood pressure (SBP) < 100 mmHg, and altered mentation were independent risk factors of 28-day mortality. We developed new screening tools named the S-TRIAGE score, which has the respiratory rate (< 11, > 22 breaths/min), the ratio of pulse oximetric saturation to the fraction of inspired oxygen (< 420, 420–450), SI (> 1, 0.6–0.99), SBP < 100 mmHg, body temperature (< 36, > 37.5 °C), and mental change. The area under a receiver operating characteristic (ROC) curve of the S-TRIAGE score in the validation cohort was 0.826 [95% confidence interval (95%CI) 0.773–0.879] in predicting mortality and lifesaving intervention, and the clinical score classified patients into five groups.

**Conclusion:**

This study showed malignancy, hypotension, increased SI, and mental status change were predictive factors for 28-day mortality in older adults in the ED. The screening tool risk score for geriatrics used in this study is potentially a good predictor of mortality and lifesaving intervention in high-risk older patients in the ED.

**Supplementary Information:**

The online version contains supplementary material available at 10.1186/s12245-023-00538-5.

## Introduction

As the world’s older population continues to grow, the number of emergency visits to hospitals by older adults is also increasing, with consequent more frequent use of emergency services and a greater level of urgency compared with younger counterparts [1]. Furthermore, with multiple comorbidities, use of polypharmacy, and potentially severe illness, older patients are at higher risk of adverse outcomes, extended hospitalization, readmission to the emergency department (ED), and mortality [2]. These situations significantly affect ED capacity and capability, leading to an urgent demand for appropriate screening tools for geriatrics-related emergency services [3].

Older patients may delay proper assessment and special care needs because they have unspecified clinical presentation. In previous studies, many triage systems demonstrated mixed performance in predicting adverse outcomes in older patients [4–7]. For example, the Emergency Severity Index (ESI), widely used in the ED, divides patients into five classification levels [8]. However, recent studies reported that ESI had poor accuracy in predicting patients who need immediate lifesaving intervention, with most older patients in the ED running the risk of under-triage [5, 6]. Several research groups developed geriatric-specific triage tools such as the Identification of Seniors at Risk and the Triage Risk Screening Tool to identify at-risk patients [9, 10]. However, the history taking of older patients might be complicated by several conditions [3]. Most studies showed these instruments to be unreliable as a single predictor of adverse outcomes [11, 12].

The National Early Warning Score (NEWS) is a simple tool calculated from vital sign parameters. The score was developed to predict clinical deterioration in the ward and has recently been used in the ED to predict admission and mortality [13]. Owing to physiological changes in older people, studies reported only low to moderate effectiveness of NEWS in predicting mortality in older patients [14, 15].

This study aimed to evaluate the predictors of 28-day mortality in older patients and developed a new risk-scoring system for geriatric triage in the ED that is practical and straightforward to use.

## Methods

### Study setting and population

A single-center, retrospective observational study was conducted at Ramathibodi Hospital, a tertiary care and university hospital in Bangkok. This study was approved by the ethics committee of Ramathibodi Hospital, Mahidol University (COA. MURA2020/1791). In addition, the Ethics Committee waived each patient’s need for informed consent.

Patients aged 65 years and older who visited the ED at Ramathibodi Hospital between January 1, 2018, and December 31, 2019, were included in this study. Patients who were in cardiac arrest at the time of arrival, trauma patients, patients who denied resuscitation, patients who transferred from the ED, and patients with missing data were excluded from the analysis. Data between January 2018 and June 2019 were used to find factors associated with 28-day mortality and construct the prediction model for mortality or receiving lifesaving interventions. Data from July 2019 was used for validating the predictive model.

### Measurement

We collected the data from the Ramathibodi Hospital database via electronic medical records. The study variables were recorded for all eligible patients, including the baseline characteristics and potential clinical risk factors for mortality. Clinical factors included age, sex, comorbidities, ESI triage, vital signs at triage (heart rate [HR], systolic blood pressure [SBP], respiratory rate [RR], oxygen saturation [SpO_2_], and body temperature [BT]), the initial fraction of inspired oxygen (FiO_2_) at triage, NEWS at ED arrival, mental change, and diagnosis.

The primary outcome aimed to find variables related to 28-day mortality. The secondary outcome was to develop a screening risk assessment score for 28-day mortality and lifesaving intervention in the ED for geriatric patients.

### Definitions

The lifesaving intervention was defined as using an invasive mechanical ventilator, noninvasive positive pressure ventilation, and the use of vasopressors in the ED. The NEWS consists of seven physiological variables: SBP, HR, RR, BT, SpO_2_, use of supplemental oxygen, and level of consciousness. Furthermore, NEWS has been categorized into four levels, and high risk is a key threshold to emergency response (Additional file 1). The ESI is a five-level ED triage algorithm based on the acuity of patients’ problems and the number of resources used. ESI level-1 indicates a patient who needs immediate lifesaving intervention. ESI level-2 is defined by a patient who has high-risk conditions of deterioration. ESI level-3 indicates a patient with an urgent condition who must use more than one resource in the ED. ESI level-4 indicates who has non-urgent conditions and needs to use only one resource in ED. Finally, ESI level-5 indicates who has non-urgent conditions and does not require using the resource in ED (Additional file 2).

### Sample size calculation

We calculated the sample size required to analyze predictor risks for 28-day mortality. Our previous hospital data on 28-day mortality in older patients who visited the ED showed 28-day mortality in 15 (4.03%) of 372 patients. Our calculation revealed that 1000 patients were required to provide an adequate sample size for the primary outcome in this study (95% confidence level, 5% alpha error).

For secondary outcome, the prevalence of lifesaving intervention in geriatrics was 5.2% [16]. We assume that the new model has eight candidate factors and approximately 25% of the variability of the Cox-Snell R squared statistic (R2cs). The sample size for the new predictive model was calculated by PMSAMPSIZE command [17] and needed 860 and 46 events for the development cohort.

### Data analyses

#### Building the prediction model

All statistical analyses were performed using Stata version 16.1 (StataCorp, College Station, TX, USA). Frequencies or percentages described the categorical variables and mean, and standard deviation (SD) or median and interquartile ranges (IQR) were used to describe continuous variables, as appropriate. Exploratory analysis was done for all potential predictors of 28-day mortality using univariable logistic regression. Odds ratio (OR) with a *P* value was reported separately for each predictor variable. Then, multivariable logistic regression with stepwise model analysis was performed to identify independent predictors of 28-day mortality. Predictor variables with a *P* value of > 0.1 were sequentially eliminated from a logistic regression model.

The measure of each variable’s performance of 28-day mortality and lifesaving intervention was reported as the area under the receiver operating characteristic curve (AUROC) with a 95% confidence interval (CI). Predictors in the new model were selected by the highest AUROC, and multicollinearity was checked by the variance inflation threshold in the new model. Each final predictor was assigned scores based on each item’s logistic regression coefficient. Finally, the scores were calculated to generate the lifesaving intervention and mortality prediction score, referred to as the “Screening Tool Risk Score Assessment in the Emergency Department for Geriatric (S-TRIAGE)” in this article.

#### Validation of the prediction score

The predictive model was evaluated for its prognostic performance in terms of discrimination and calibration. A measure of discrimination was reported as AUROC. A measure of calibration was reported as Hosmer–Lemeshow goodness of fit statistics. The cutoff prediction scores for 28-day mortality and requiring lifesaving intervention were determined using sensitivity, specificity, and predictive probability. A calibration plot comparing score predicted risk versus observed risk was presented with 1000 replication bootstrapping. The predictive score was validated with internal validation and compared with the development set by chi-square. A *P* value of < 0.05 was considered to indicate statistical significance.

## Results

During the study period, 1580 patients were eligible for the inclusion criteria, 187 of whom were excluded by meeting the exclusion criteria (2 patients with cardiac arrest, 59 trauma patients, 70 patients were transferred to other hospitals, 54 patients did not resuscitate, 2 patients with incomplete data), leaving 1393 patients for analysis. In the development cohort, among these 1002 patients, 103 (10.3%) died within 28 days after visiting the ED. Univariable analysis showed male, malignancy, sepsis, SBP, RR, HR, SpO_2_, SI, the ratio of pulse oximetric saturation to the fraction of inspired oxygen (SF ratio), and mental change were significant independent factors in patients who died within 28 days (Table 1). Under a multivariable analysis, the remaining potential factors that significantly increased mortality were: malignancy (adjusted odds ratio [aOR] = 2.50, 95% CI = 1.55–4.03, *P* =  < 0.001), SBP < 100 mmHg (aOR = 2.27, 95% CI = 1.02–5.05, *P* = 0.045), SI (aOR = 2.26, 95% CI = 1.04–1.26, *P* = 0.007), and mental change (aOR = 8.07, 95% CI = 4.25–15.33, *P* =  < 0.001) (Table 2).

**Table 1:** Baseline characteristics and univariable logistic regression of factors associated with 28-day mortality in older patients

Variable	Survivors (*N* = 899)	Non-survivors (*N* = 103)	OR (95% CI)	*p* value
Male sex, *n* (%)	401 (44.6)	57 (55.3)	1.54 (1.02–2.32)	0.039
Age (years), median (IQR)	76 (70–82)	70 (78–84)	1.02 (0.99–1.05)	0.062
Comorbidities, *n* (%)				
Hypertension	581 (64.6)	63 (61.2)	0.86 (0.57–1.31)	0.488
Diabetes mellitus	321 (35.7)	32 (31.1)	0.81 (0.52–1.26)	0.351
Atrial fibrillation	109 (12.1)	18 (17.5)	1.53 (0.89–2.65)	0.124
Coronary artery disease	166 (18.5)	25 (24.3)	1.42 (0.87–2.29)	0.157
Chronic obstructive pulmonary disease and asthma	93 (10.3)	16 (15.5)	1.59 (0.90–2.83)	0.112
All-stage of malignancy disease	203 (22.6)	46 (44.7)	2.77 (1.82–4.21)	< 0.001
Chronic kidney disease stages 4, 5	70 (7.8)	11 (10.7)	1.42 (0.72–2.77)	0.310
Old cerebrovascular accident	125 (13.9)	13 (12.6)	0.89 (0.49–1.65)	0.721
Neurodegenerative disease	68 (7.6)	5 (4.9)	0.62 (0.25–1.58)	0.326
Diagnosis: sepsis, *n* (%)	95 (10.6)	28 (27.2)	3.16 (1.94–5.12)	< 0.001
Vital signs, median (IQR)				
Systolic blood pressure, mmHg	143 (125–166)	125 (104–148)	0.98 (0.97–0.99)	< 0.001
Respiratory rate, breaths/min	20 (20–22)	22 (20–26)	1.12 (1.07–1.16)	< 0.001
Heart rate, bpm	87 (73–100)	98 (80–112)	1.02 (1.00–1.03)	< 0.001
Body temperature, °C	37 (36.5–37.5)	37.1 (36.6–38.1)	1.22 (0.98–1.52)	0.074
SpO_2_, %	96 (96–97)	96 (96–98)	0.94 (0.90–0.98)	0.009
Shock index, mean (SD)	0.64 ± 0.23	0.80 ± 0.26	1.28 (1.18–1.38)	< 0.001
SF ratio, median (IQR)	457 (457–461)	457 (433–467)	0.99 (0.98–0.99)	< 0.001
Mental change, *n* (%)	30 (3.3)	30 (25.6)	11.90 (6.80–20.83)	< 0.001
NEWS, median (IQR)	1 (1–4)	5 (2–7)	1.32 (1.25–1.41)	< 0.001
ESI, median (IQR)	3 (3–3)	2 (2–2)	0.26 (0.16–0.41)	< 0.001

*OR* odd ratio, *95% CI* 95% confidence interval, *IQR* interquartile range, *bpm* beats per minute, *SpO*_*2*_ oxygen saturation, *SD* standard deviation, *SF ratio* ratio of the pulse oximetric saturation to the fraction of inspired oxygen, *NEWS* National Early Warning Score, *ESI* Emergency Severity Index

**Table 2:** Multivariable logistic regression by stepwise model of factors associated with 28-day mortality in older patients

Variable	Adjusted odd ratio (95%CI)	*p* value
Age (per year)	1.03 (0.99–1.06)	0.067
Chronic kidney disease stages 4, 5	1.79 (0.89–4.01)	0.126
Neurodegenerative disease	0.44 (0.15–1.27)	0.127
All-stage of malignancy disease	2.50 (1.55–4.03)	< 0.001
Shock (SBP < 100 mmHg)	2.27 (1.02–5.05)	0.045
Shock index (per increase 0.1)	2.26 (1.04–1.26)	0.007
Respiratory rate (per breath/min)	1.05 (0.99–1.10)	0.090
Mental status change	8.07 (4.25–15.33)	< 0.001

*95% CI* 95% confidence interval, *SBP* systolic blood pressure

The general characteristics of the study population between the development and validation cohort were summarized in Table 3. Furthermore, the performance of each variable to predict mortality and lifesaving intervention in the development cohort was shown in Table 4, and the six highest AUROC predictors were selected to develop the new model.

**Table 3:** Baseline characteristics between the development cohort and validation cohort

Variable	Development cohort (*N* = 1002)	Validation cohort (*N* = 391)	*p* value
Male sex, *n* (%)	458 (45.71)	183 (46.8)	0.713
Age (years), median (IQR)	77 (70,75)	77 (71,83)	0.264
Comorbidities, *n* (%)			
Hypertension	644 (64.3)	240 (61.4)	0.314
Diabetes mellitus	353 (35.2)	159 (40.6)	0.059
Atrial fibrillation	127 (12.7)	35 (8.9)	0.051
Coronary artery disease	191 (19.1)	87 (22.2)	0.181
Chronic obstructive pulmonary disease and asthma	109 (10.9)	37 (9.4)	0.439
All-stage of malignancy disease	249 (24.9)	99 (25.3)	0.856
Chronic kidney disease stages 4, 5	81 (8.1)	98 (25.1)	< 0.001
Old cerebrovascular accident	138 (13.8)	50 (12.7)	0.629
Neurodegenerative disease	73 (7.3)	35 (8.9)	0.296
Diagnosis: sepsis, *n* (%)	123 (12.3)	39 (10.0)	0.229
Vital signs, median (IQR)			
Systolic blood pressure, mmHg	142 (122–165)	137 (122–163)	0.286
Respiratory rate, breaths/min	20 (20–24)	20 (20–22)	0.038
Heart rate, bpm	88 (73–102)	87 (74–100)	0.821
Body temperature, °C	37 (36.5–37.6)	36.7 (36.3–37.2)	< 0.001
SpO_2_, %	96 (96–97)	98 (96–99)	< 0.001
Shock index, mean (SD)	0.66 ± 0.23	0.66 ± 0.23	0.656
SF ratio, median (IQR)	457 (457–461)	466 (457–471)	< 0.001
Mental change, *n* (%)	60 (5.9)	48 (12.2)	< 0.001

*IQR* interquartile range; *bpm*, beat per minute, *SpO*_*2*_ oxygen saturation, *SD* standard deviation, *SF* ratio ratio of the pulse oximetric saturation to the fraction of inspired oxygen

**Table 4:** Performance of variables for predicting mortality and intervention in older patients in development cohort (*N* = 1002)

Variable	AUROC (95%CI)	OR (95% CI)	*p* value
Male sex	0.508 (0.467–0.548)	1.06 (0.77–1.47)	0.709
Age (years)	0.592 (0.547–0.637)	1.04 (1.02–1.06)	< 0.001
Comorbidities			
Hypertension	0.489 (0.450–0.527)	0.91 (0.65–1.27)	0.568
Diabetes mellitus	0.508 (0.469–0.546)	1.07 (0.76–1.49)	0.701
Atrial fibrillation	0.541 (0.510–0.571)	1.90 (1.24–2.91)	0.003
Coronary artery disease	0.535 (0.501–0.570)	1.53 (1.04–2.24)	0.029
Chronic obstructive pulmonary disease and asthma	0.551 (0.521–0.582)	2.42 (1.56–3.76)	< 0.001
All-stage of malignancy disease	0.561 (0.523–0.598)	1.82 (1.29–2.58)	0.001
Chronic kidney disease stages 4, 5	0.528 (0.502–0.554)	1.91 (1.15–3.20)	0.013
Old cerebrovascular accident	0.537 (0.506–0.569)	1.75 (1.15–2.66)	0.009
Neurodegenerative disease	0.510 (0.487–0.532)	1.30 (0.73–2.32)	0.375
Diagnosis: sepsis	0.600 (0.566–0.635)	4.26 (2.84–6.38)	< 0.001
Vital signs			
Systolic blood pressure, mmHg	0.611 (0.563–0.660)	0.99 (0.98–0.99)	< 0.001
Respiratory rate, breaths/min	0.754 (0.713–0.795)	1.28 (1.22–1.34)	< 0.001
Heart rate, bpm	0.650 (0.605–0.696)	1.02 (1.01–1.03)	< 0.001
Body temperature, °C	0.578 (0.530–0.626)	1.34 (1.12–1.59)	0.001
SpO_2_, %	0.572 (0.517–0.623)	0.85 (0.81–0.89)	< 0.001
Shock index	0.675 (0.630–0.720)	1.28 (1.20–1.36)	< 0.001
SF ratio	0.625 (0.571–0.679)	0.99 (0.98–0.99)	< 0.001
Mental change	0.601 (0.571–0.633)	12.36 (6.97–21.92)	< 0.001

*AUROC* area under the receiving operating characteristic curve, *95% CI* 95% confidence interval, *OR* odds ratio, *bpm*, beat per minute, *SpO*_*2*_ oxygen saturation, *SF ratio* ratio of the pulse oximetric saturation to the fraction of inspired oxygen

The item score was developed by significant vital signs with the RR (< 11, > 22 breaths/min), SF ratio (≤ 420, 420–450), SI (0.6–0.99, ≥ 1), SBP (< 100 mmHg), BT (< 36 or > 37.5 °C), altered mentation and the score of each predictive factor was shown in Table 5. The prediction score was named “Screening Tool Risk Score Assessment in the Emergency Department for Geriatric (S-TRIAGE)” score.

**Table 5:** Development of scoring to predict mortality and receiving intervention in older patients in the development cohort (*N* = 1002)

Variables	Coefficient	Adjusted OR (95% CI)	*p* value	Score
Respiratory rate				
< 11 or > 22	1.36	3.91 (2.56–5.97)	< 0.001	5
SF ratio				
420 < x < 450	1.00	2.72 (1.54–4.78)	0.001	4
≤ 420	1.28	3.59 (1.86–6.91)	< 0.001	5
Shock index				
0.6 − 0.99	0.40	1.48 (0.97–4.78)	0.072	1.5
≥ 1	0.72	3.59 (1.86–6.91)	0.048	3
SBP < 100	1.13	3.10 (1.44–6.66)	0.004	4
Body temperature				
< 36 or > 37.5 C	0.27	1.32 (0.87–2.00)	0.189	1
Mental change	1.68	5.4 (2.69–10.85)	< 0.001	6
Total S-TRIAGE score				24

Hosmer–Lemeshow *P* value = 0.618

*OR* odds ratio, *95% CI* 95% confidence interval, *SBP* systolic blood pressure, *S-TRIAGE* Screening Tool Risk Score Assessment in the Emergency Department for Geriatric

In the development cohort, the predictive model was divided into five-level to predict mortality and receiving lifesaving intervention: very high risk (score > 10; sensitivity 40.3% and specificity 95.2%), high risk (score > 7 or ≤ 10; sensitivity 56.9% and specificity 87.3%), moderate risk (score > 3 or ≤ 7; sensitivity 79.0% and specificity 74.2%), low risk (score > 1 or ≤ 3; sensitivity 91.2% and specificity 42.3%), and very low risk (score 0–1). The mortality and lifesaving intervention probability in both cohorts are shown in Table 6.

**Table 6:** Performance of S-TRIAGE score to predict mortality and receiving intervention in older patients in the development cohort and validation cohort

Scoring	Sensitivity (95%CI)	Specificity (95%CI)	PPV (95%CI)	NPV (95%CI)	Probability (95%CI)
Development cohort (*N* = 1002) S-TRIAGE score; AUROC = 0.824 (0.789–0.859)					
0–1	-	-	-	-	4.4% (2.3–6.5%)
1 < score < = 3	91.2% (86.0–94.9%)	42.3% (38.9–45.7%)	25.8% (22.5–29.4%)	95.6% (92.9–97.5%)	7.7% (4.6–10.9%)
3 < score < = 7	79.0% (72.3–84.7%)	74.2% (71.0–77.1%)	40.3% (35.1–45.6%)	94.1% (92.0–95.8%)	27.0% (19.9–34.2%)
7 < score < = 10	56.9% (49.4–64.2%)	87.3% (84.9–89.5%)	49.8% (42.8–56.8%)	90.2% (87.9–92.2%)	31.6% (22.2–40.9%)
> 10	40.3% (33.1–47.9%)	95.2% (93.6–96.6%)	65.2% (55.6–73.9%)	87.9% (85.5–89.9%)	65.2% (56.4–74.0%)
Validation cohort (*N* = 391) S-TRIAGE score; AUROC = 0.826 (0.773–0.879)					
0–1	-	-	-	-	3.8% (0.5–6.9%)
1 < score < = 3	93.2% (84.7–97.7%)	40.3% (34.8–45.9%)	26.4% (21.1–32.2%)	96.2% (91.4–98.8%)	5.6% (0.8–10.3%)
3 < score < = 7	86.3% (76.2–93.2%)	67.0% (61.5–72.1%)	37.5% (30.2–45.3%)	95.5% (91.9–97.8%)	22.2% (12.0–32.5%)
7 < score < = 10	67.1% (55.1–77.7%)	82.4% (77.7–86.4%)	46.7% (36.9–56.7%)	91.6% (87.8–94.5%)	36.6% (21.8–51.3%)
> 10	46.6% (34.8–58.6%)	90.6% (86.8–93.5%)	53.1% (40.2–65.7%)	88.1% (84.1–91.4%)	53.1% (40.9–65.4%)

*S-TRIAGE* Screening Tool Risk Score Assessment in the Emergency Department for Geriatric, *95% CI* 95% confidence interval, *PPV* positive predictive value, *NPV* negative predictive value, *AUROC* area under the receiving operating characteristic curve

Our study realized an AUROC 0.824 (95% CI = 0.789–0.859) in the development cohort and 0.826 (95% CI = 0.773–0.879) in the validation cohort (AUROC different − 0.002, *P* = 0.955, Table 7) for the ability of the S-TRIAGE score to predict 28-day mortality and receiving the lifesaving intervention. In addition, the calibration of the risk score showed the predicted risk and observed risk of outcomes in elderly patients, presented in Additional files 3–4. Moreover, the S-TRIAGE score had a better performance than NEWS and ESI in the development cohort, as shown by the AUROC difference (*P* = 0.105 and < 0.001, respectively, Additional file 5).

**Table 7:** Comparison of AUROC of the scoring system between development cohort and validation cohort

Scoring	Development (*N* = 1002)	Validation (*N* = 391)	AUROC different	*p* value
S-TRIAGE score	0.824 (0.789–0.859)	0.826 (0.773–0.879)	− 0.002	0.955
NEWS	0.808 (0.771–0.847)	0.830 (0.775–0.885)	− 0.032	0.535
ESI	0.706 (0.671–0.740)	0.781 (0.730–0.833)	− 0.075	0.016

*AUROC* area under the receiving operating characteristic curve, *S-TRIAGE* Screening Tool Risk Score Assessment in the Emergency Department for Geriatric, *NEWS* National Early Warning Score, *ESI* Emergency Severity Index

We suggested that a score > 7 and ≤ 10 points represented a danger zone, whereby emergency care should trigger tools for closely monitoring this patient group. Additionally, patients in this very high-risk group (score > 10) would need lifesaving intervention or a high level of care, such as admission to the intensive care unit (ICU).

## Discussion

This study assessed variables associated with mortality and developed a screening tool risk score to predict mortality and requiring lifesaving intervention for geriatric patients in the ED. The result showed malignancy, SBP < 100 mmHg, increased shock index, and mental status change as potential predictors of mortality in older patients. We developed the S-TRIAGE score from RR, SF ratio, SI, SBP < 100mmHg, BT, and altered mentation, which is suitable in the triage area—whereby the cutoff of each parameter was not the same as that of the general population. The S-TRIAGE score performed well in predicting 28-day mortality and the need for lifesaving intervention in the ED. In applying the clinical score that classified patients into five groups, we suggest consideration of close monitoring of high-risk patients while patients at very high risk require resuscitation and a high level of care. In addition, we propose that S-TRIAGE combined with ESI triage may increase the precision of predicting triage level 1, i.e., the immediate requirement of lifesaving intervention or triage level 2 for decision patients with high-risk conditions of deterioration.

Vital physiological signs are commonly used to assess a patient’s condition for a medical emergency, such as ESI triage and Early Warning Score for an activated rapid response team. However, physiological variables may be significantly affected by age. Therefore, our study indicated for each parameter a new cutoff suitable for predicting geriatric patients at risk.

Older patients had rapid RR to compensate for low tidal volume attributable to a decline in lung elastic recoil [18], so a low RR represented respiratory failure. Moreover, Smith et al. reported that a breathing rate > 24 breaths/min in older patients was associated with mortality [19].

The SF ratio was used as a non-invasive and alternative marker of the PaO_2_/FiO_2_ratio in acute respiratory failure and was significantly associated with mortality in ICU [20]. Similarly, Kwack et al. reported that the SF ratio was a significant factor in predicting transfer to the ICU and mortality [21].

Age-related molecular change in cardiovascular systems results in sensitivity, reliability, and normal blood pressure and HR range [18, 22]. SI is a ratio of HR over SBP that is easily calculated in the ED. Previous studies revealed that SI was related to high mortality and the need for life-saving intervention in geriatric patients [23–25]. Also, our study showed SBP was a significant factor in predicting mortality, with the trigger cutoff SBP indicating low blood pressure of < 100 mmHg. Compared with the results reported by Smith et al., older patients with SBP in the 90–94 mmHg range had ten times the mortality of younger counterparts [19].

This study confirms that low BT and fever were related to mortality in older patients. Fever was considered an indicator of immune response for sepsis and inflammation [26], while hypothermia represented severe conditions and multiorgan failure [27]. However, the cutoff BT in the elderly was lower than in the younger because of the reduced production of pyrogenic cytokines and changed thermoregulation by aging [18].

Acute changes in mental status are usually precipitated by an underlying medical illness that is potentially life-threatening [28]. Our data showed that altered mentation was a potential factor for predicting mortality in older patients. Moreover, Boonmee et al. found that Glasgow Coma Scale scores were associated with mortality in sepsis in older adults [29].

The S-TRIAGE score achieves excellent performance in terms of mortality and requiring lifesaving intervention prediction, while ESI triage performed low accuracy on both the development and validation cohort. Nevertheless, the ability of NEWS to predict mortality and lifesaving intervention in this validation cohort was also favorable but performed less well in the development cohort. Comparing this result with previous studies, the predictive value of NEWS in older patients in the ED regarding mortality remains to be determined [14, 15].

This study had several limitations. First, it was a retrospective single-center study, which reduces its generalizability to broader populations. Second, as ESI triage was a decision of triage nursing staff whose degree of experience affected the accuracy of triage, some patients may have been under-triage, thus limiting the usefulness of ESI in this study. Third, because the cutoff point of each parameter was selected from data for this cohort, the results achieved a good performance level. Further studies are needed for external validation and to remove selection bias.

## Conclusions

This study showed malignancy, SBP < 100 mmHg, increased shock index, and mental status change were predictive factors for 28-day mortality in elderly patients in the ED. In addition, our newly developed screening tool, the S-TRIAGE score, was simple and performed well in stratifying patients at risk who need close monitoring and resuscitation.

### Supplementary Information

**Additional file 1.** The National Early Warning Score (NEWS) Clinical Parameters and Classification.

**Additional file 2.** Emergency Severity Index (ESI) Triage Classification and Description.

**Additional file 3.** Calibration graph of STRAIGE score in 1,000 bootstrapping in development cohort.

**Additional file 4.** Calibration graph of S-TRAIGE score in 1,000 bootstrapping in validation cohort.

**Additional file 5.** Comparison of AUROC of scoring system for predicting mortality and receiving intervention in elderly patients in development cohort.

## Data Availability

The datasets analyzed in this study are not publicly available owing to privacy issues but are available from the corresponding author upon reasonable request.

## Abbreviations

aOR: Adjusted odds ratio
AUROC: Area under the receiver operating characteristic curve
BT: Body temperature
CI: Confidence level
ED: Emergency department
ESI: Emergency Severity Index
FiO_2_: Initial fraction of inspired oxygen
HR: Heart rate
ICU: Intensive care unit
IQR: Interquartile range
NEWS: The National Early Warning Score
RR: Respiratory rate
SBP: Systolic blood pressure
SF ratio: The ratio of pulse oximetric saturation to the fraction of inspired oxygen
SI: Shock index
SpO_2_: Oxygen saturation
S-TRIAGE: Screening Tool Risk Score Assessment in the Emergency Department for Geriatric

## References

[1] Roberts DC, McKay MP, Shaffer A (2008). Increasing rates of emergency department visits for elderly patients in the United States, 1993 to 2003. Ann Emerg Med.

[2] Aminzadeh F, Dalziel WB (2002). Older adults in the emergency department: a systematic review of patterns of use, adverse outcomes, and effectiveness of interventions. Ann Emerg Med.

[3] Salvi F, Morichi V, Grilli A, Giorgi R, De Tommaso G, Dessì-Fulgheri P (2007). The elderly in the emergency department: a critical review of problems and solutions. Intern Emerg Med.

[4] Baumann MR, Strout TD (2007). Triage of geriatric patients in the emergency department: validity and survival with the emergency severity index. Ann Emerg Med.

[5] Grossmann FF, Zumbrunn T, Frauchiger A, Delport K, Bingisser R, Nickel CH (2012). At risk of undertriage? Testing the performance and accuracy of the emergency severity index in older emergency department patients. Ann Emerg Med.

[6] Platts-Mills TF, Travers D, Biese K, McCall B, Kizer S, Lamantia M (2010). Accuracy of the emergency severity index triage instrument for identifying elder emergency department patients receiving an immediate life-saving intervention. Acad Emerg Med.

[7] Brouns SHA, Mignot-Evers L, Derkx F, Lambooij SL, Dieleman JP, Haak HR (2019). Performance of the Manchester triage system in older emergency department patients: a retrospective cohort study. BMC Emerg Med.

[8] Gilboy N, Tanabe P, Travers DA, Rosennau AM, Eitel DR (2011). Emergency Severity Index (ESI): a triage tool for emergency department.

[9] Tavares JPA, Sa-Couto P, Boltz M, Capezuti E (2017). Identification of Seniors at Risk (ISAR) in the emergency room: a prospective study. Int Emerg Nurs.

[10] Rizka A, Ambarwati RA, Mansjoer A, Koesnoe S (2021). Performance of Identification of the Seniors at Risk (ISAR) tool and Triage Risk-Screening Tool (TRST) for frail elderly emergency room patients. J Pak Med Assoc.

[11] Buurman BM, van den Berg W, Korevaar JC, Milisen K, de Haan RJ, de Rooij SE (2011). Risk for poor outcomes in older patients discharged from an emergency department: feasibility of four screening instruments. Eur J Emerg Med.

[12] Pham KD, Lim FA (2020). The impact of geriatric-specific triage tools among older adults in the emergency department. Crit Care Nurs Q.

[13] Alam N, Vegting IL, Houben E, Berkel BV, Vaughan L, Kramer MH (2015). Exploring the performance of the National Early Warning Score (NEWS) in a European emergency department. Resuscitation.

[14] Kemp K, Alakare J, Harjola V-P, Strandberg T, Tolonen J, Lehtonen L (2020). National Early Warning Score 2 (NEWS2) and 3-level triage scale as risk predictors in frail older adults in the emergency department. BMC Emerg Med.

[15] Kim I, Song H, Kim HJ, Park KN, Kim SH, Oh SH (2020). Use of the National Early Warning Score for predicting in-hospital mortality in older adults admitted to the emergency department. Clin Exp Emerg Med.

[16] Lee JY, Oh SH, Peck EH, Lee JM, Park KN, Kim SH (2011). The validity of the Canadian triage and acuity scale in predicting resource utilization and the need for immediate life-saving interventions in elderly emergency department patients. Scand J Trauma Resusc Emerg Med.

[17] Riley RD, Ensor J, Snell KIE, Harrell FE, Martin GP, Reitsma JB (2020). Calculating the sample size required for developing a clinical prediction model. BMJ..

[18] Chester JG, Rudolph JL (2011). Vital signs in older patients: age-related changes. J Am Med Dir Assoc.

[19] Smith GB, Prytherch DR, Schmidt PE, Featherstone PI, Kellett J, Deane B (2008). Should age be included as a component of track and trigger systems used to identify sick adult patients?. Resuscitation.

[20] Fukuda Y, Tanaka A, Homma T, Kaneko K, Uno T, Fujiwara A (2021). Utility of SpO2/FiO2 ratio for acute hypoxemic respiratory failure with bilateral opacities in the ICU. PLoS One.

[21] Kwack WG, Lee DS, Min H, Choi YY, Yun M, Kim Y (2018). Evaluation of the SpO2/FiO2 ratio as a predictor of intensive care unit transfers in respiratory ward patients for whom the rapid response system has been activated. PLoS One.

[22] Molander L, Lovheim H, Norman T, Nordström P, Gustafson Y (2008). Lower systolic blood pressure is associated with greater mortality in people aged 85 and older. J Am Geriatr Soc.

[23] Tzadok B, Soffer S, Shapira S, Or ET (2019). Shock index in the acute care geriatric patient and its correlation to the need of a life saving intervention. Int J Gerontol Geriatr Res.

[24] Pandit V, Rhee P, Hashmi A, Kulvatunyou N, Tang A, Khalil M (2014). Shock index predicts mortality in geriatric trauma patients: an analysis of the National Trauma Data Bank. J Trauma Acute Care Surg.

[25] Chung JY, Hsu CC, Chen JH, Chen WL, Lin HJ, Guo HR (2019). Shock index predicted mortality in geriatric patients with influenza in the emergency department. Am J Emerg Med.

[26] Shimazui T, Nakada TA, Walley KR, Oshima T, Abe T, Ogura H (2020). Significance of body temperature in elderly patients with sepsis. Crit Care.

[27] Alakare J, Kemp K, Strandberg T, Castrén M, Tolonen J, Harjola VP (2022). Low body temperature and mortality in older patients with frailty in the emergency department. Aging Clin Exp Res.

[28] Han JH, Wilber ST (2013). Altered mental status in older patients in the emergency department. Clin Geriatr Med.

[29] Boonmee P, Ruangsomboon O, Limsuwat C, Chakorn T (2020). Predictors of mortality in elderly and very elderly emergency patients with sepsis: a retrospective study. West J Emerg Med.

