# Supplementary material for: Screening Tool Risk Score Assessment in the Emergency Department for Geriatric (S-TRIAGE) in 28-day mortality
Source: Int J Emerg Med. 2023 Sep 26;16:60. doi: 10.1186/s12245-023-00538-5 (PMC10521457; doi:10.1186/s12245-023-00538-5)
Supplement: Supplementary file 1 — Additional file 1. The National Early Warning Score (NEWS) Clinical Parameters and Classification. [file 12245_2023_538_MOESM1_ESM.docx]

**Additional file 1.** The National Early Warning Score (NEWS) Clinical Parameters and Classification.

| **Physiological Parameters** | **Score** | | | | | | |
| --- | --- | --- | --- | --- | --- | --- | --- |
|  | 3 | 2 | 1 | 0 | 1 | 2 | 3 |
| Respiratory rate, breath/minute | ≤8 |  | 9-11 | 12-20 |  | 21-24 | ≥25 |
| SpO2 scale 1 (%) | ≤91 | 92-93 | 94-95 | ≥96 |  |  |  |
| SpO2 scale 2 (%) | ≤83 | 84-85 | 86-87 | 88-92  ≥93 on air | 93-94 on oxygen | 95-96 on oxygen | ≥97 on oxygen |
| Air or oxygen? |  | Oxygen |  | Air |  |  |  |
| Systolic blood pressure, mmHg | ≤90 | 91-100 | 101-110 | 111-219 |  |  | ≥220 |
| Pulse, bpm | ≤40 |  | 41-50 | 51-90 | 91-110 | 111-130 | ≥131 |
| Consciousness |  |  |  | Alert |  |  | CVPU |
| Temperature (°C) | ≤35.0 |  | 35.1-36.0 | 36.1-38.0 | 38.1-39.0 | ≥39.1 |  |
| **Total NEWS score** | 20 | | | | | | |
| **Clinical risk** | | | Low | | Aggregate score 0-4 | | |
|  |  |  | Low-medium | | Red score (Score of 3 in any individual parameter) | | |
|  |  |  | Medium | | Aggregate score 5-6 | | |
|  |  |  | High | | Aggregate score 7 or more | | |

SpO2, oxygen saturation; bpm, beat per minute
