# Supplementary material for: Screening Tool Risk Score Assessment in the Emergency Department for Geriatric (S-TRIAGE) in 28-day mortality
Source: Int J Emerg Med. 2023 Sep 26;16:60. doi: 10.1186/s12245-023-00538-5 (PMC10521457; doi:10.1186/s12245-023-00538-5)
Supplement: Supplementary file 2 — Additional file 2. Emergency Severity Index (ESI) Triage Classification and Description. [file 12245_2023_538_MOESM2_ESM.docx]

**Additional file 2.** Emergency Severity Index (ESI) Triage Classification and Description.

| ESI triage | |
| --- | --- |
| Level | **Description** |
| 1 | Require immediate life-saving intervention |
| 2 | High risk situation or confused/lethargic/disoriented or severe pain/distress |
| 3 | Require many ED resources |
| 4 | Require one ED resource |
| 5 | Not require any resources |

ED, emergency department.
