# Supplementary figures and images for: Screening Tool Risk Score Assessment in the Emergency Department for Geriatric (S-TRIAGE) in 28-day mortality
Source: Int J Emerg Med. 2023 Sep 26;16:60. doi: 10.1186/s12245-023-00538-5 (PMC10521457; doi:10.1186/s12245-023-00538-5)

**Additional file 3.** Calibration graph of S-TRAIGE score in 1,000 bootstrapping in development cohort.

**
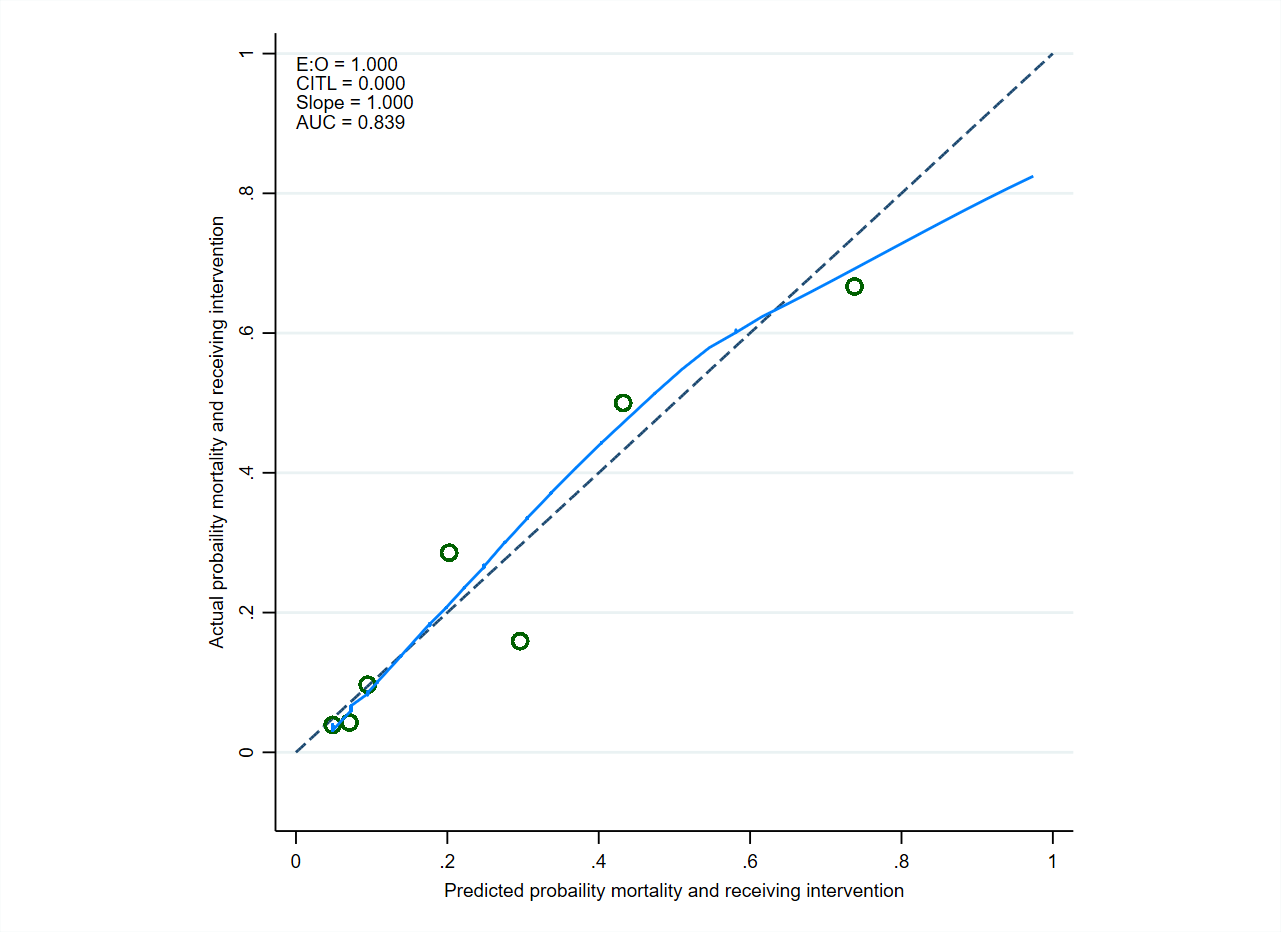
**

Supplement: Supplementary file 3 — Additional file 3. Calibration graph of STRAIGE score in 1,000 bootstrapping in development cohort. [file 12245_2023_538_MOESM3_ESM.docx]

**Additional file 4.** Calibration graph of S-TRAIGE score in 1,000 bootstrapping in validation cohort.

**
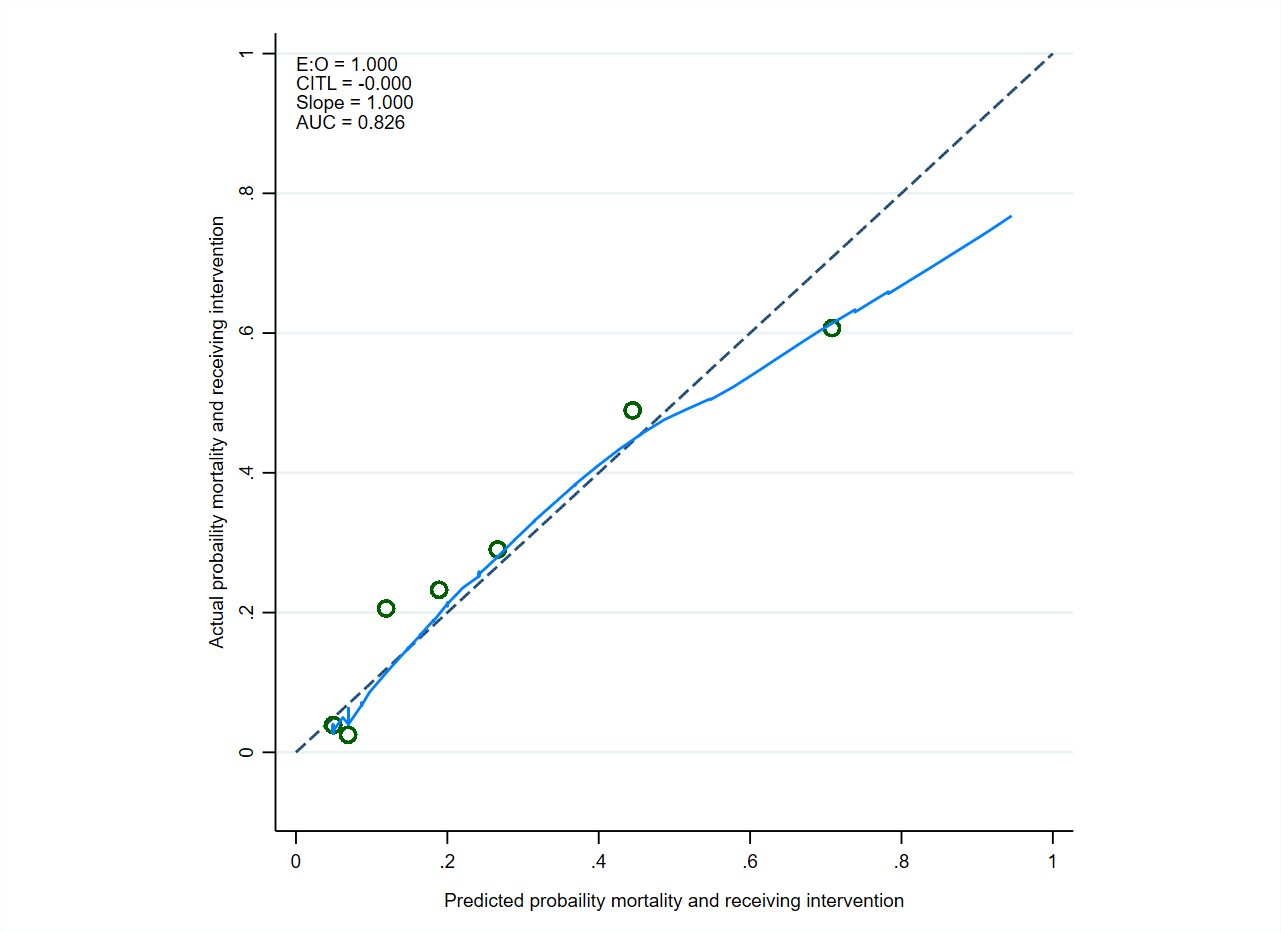
**

Supplement: Supplementary file 4 — Additional file 4. Calibration graph of S-TRAIGE score in 1,000 bootstrapping in validation cohort. [file 12245_2023_538_MOESM4_ESM.docx]
