# Supplementary material for: Screening Tool Risk Score Assessment in the Emergency Department for Geriatric (S-TRIAGE) in 28-day mortality
Source: Int J Emerg Med. 2023 Sep 26;16:60. doi: 10.1186/s12245-023-00538-5 (PMC10521457; doi:10.1186/s12245-023-00538-5)
Supplement: Supplementary file 5 — Additional file 5. Comparison of AUROC of scoring system for predicting mortality and receiving intervention in elderly patients in development cohort. [file 12245_2023_538_MOESM5_ESM.docx]

**Additional file 5.** Comparison of AUROC of scoring system for predicting mortality and receiving intervention in elderly patients in development cohort.

| Scoring | AUROC  (95% CI) | AUROC different with S-TRIAGE  p-value | AUROC different with NEWS  p-value | AUROC different with ESI  p-value |
| --- | --- | --- | --- | --- |
| S-TRIAGE | 0.824  (0.789-0.859) | - | 0.016  p-value 0.105 | 0.118  p-value <0.001 |
| NEWS | 0.808  (0.771-0.847) | 0.016  p-value 0.105 | - | 0.102  p-value <0.001 |
| ESI | 0.706  (0.671-0.740) | 0.118  p-value <0.001 | 0.102  p-value <0.001 | - |

AUROC, area under receiver operating characteristic curve; 95% CI, 95% confidence interval; S-TRIAGE, Screening Tool Risk Score Assessment in the Emergency Department for Geriatric; NEWS, National early warning score; ESI, emergency severity index
